# Five Questions about Non-Mevalonate Isoprenoid Biosynthesis

**DOI:** 10.1371/journal.ppat.1002323

**Published:** 2011-12-22

**Authors:** Audrey R. Odom

**Affiliations:** Departments of Pediatrics and Molecular Microbiology, Washington University School of Medicine, St. Louis, Missouri, United States of America; University of Wisconsin Medical School, United States of America

## What Is an Isoprenoid?

**Figure 1:**
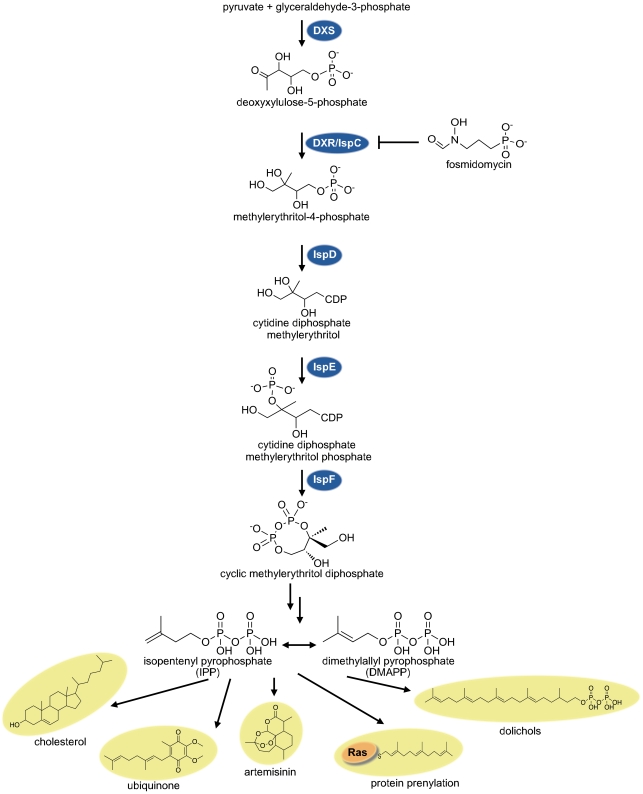
The non-mevalonate MEP pathway of isoprenoid biosynthesis. Isoprenoids are derived from the basic 5-carbon isoprenoid building blocks isopentenyl pyrophosphate (IPP) and its isomer, dimethylallyl pyrophosphate (DMAPP). In the MEP pathway, IPP and DMAPP are generated from pyruvate and glyceraldehyde 3-phosphate. Enzymes of this pathway are named here according to their *E. coli* homologs (DXR/IspC, IspD, IspE, and IspF). Fosmidomycin is a phosphonic acid antibiotic (PubChem compound ID 572) that inhibits a rate-limiting enzyme of this pathway (DXR/IspC) and blocks isoprenoid biosynthesis in vivo. Isoprenoids have great diversity in structure and cellular function—from plasma membrane stability (cholesterol), electron transport (ubiquinone), and cell wall biosynthesis (dolichols) to protein modification (as prenyl groups) and secondary metabolites (such as artemisinin).

Isoprenoids (also referred to as terpenoids) are the largest group of natural products, comprising over 25,000 known compounds [1]. Each member of this class is assembled from 5-carbon (C_5_) isoprene units and derived metabolically from the basic building block isopentenyl pyrophosphate (IPP) and its isomer, dimethylallyl pyrophosphate (DMAPP). Subsequent metabolic reactions (such as cyclization) generate enormous complexity and diversity from these basic starting materials. Isoprenoids are vital to all organismal classes, supporting core cellular functions such as aerobic respiration (ubiquinones) and membrane stability (cholesterol) (Figure 1). Isoprenoids also form the largest group of so-called secondary metabolites, such as the extremely diverse classes of plant defensive terpenoids that are widely exploited as perfumes, food additives, and pharmaceutical agents (e.g., the antimalarial compound artemisinin) [2].

## What Is the Non-Mevalonate (MEP) Pathway of Isoprenoid Biosynthesis?

The basic isoprenoid building blocks (IPP and DMAPP) are generated in cells by one of two distinct biosynthetic routes. The classical mevalonate (MVA) pathway was first described in yeast and mammals in the 1950s [3]. IPP is synthesized from acetyl-CoA via the key metabolite mevalonate (MVA). The widely used “statin” class of cholesterol-lowering drugs targets the rate-limiting enzyme of the MVA pathway, HMG-CoA reductase.

By the early 1990s, however, the existence of an alternative route to IPP had become clear through metabolic labeling studies in bacteria and plants [4]. This alternate “non-mevalonate” pathway does not share any enzymes or metabolites with the MVA pathway. It begins by generation of deoxyxyluose 5-phosphate (DOXP) from pyruvate and glyceraldehyde 3-phosphate. The first dedicated metabolite of this pathway, methylerythritol 4-phosphate (MEP)—from which the pathway derives its name—is then generated from DOXP by deoxyxylulose phosphate reductoisomerase (DXR, also known as IspC). Subsequent enzymatic steps convert MEP to IPP/DMAPP for synthesis of downstream isoprenoids. Since the MEP pathway is linear, each of the enzymes in this pathway is required for de novo isoprenoid biosynthesis.

## Which Microbes Use the MEP Pathway?

The two isoprenoid biosynthetic routes, the mevalonate (MVA) and non-mevalonate (MEP) pathways, have distinct evolutionary origins and are phylogenetically compartmentalized [5], [6]. Archaebacteria and most eukaryotes, including all metazoans and fungi, use the MVA pathway. In contrast, the overwhelming majority of eubacteria use the MEP pathway, including key pathogens such as all Gram-negative bacteria and mycobacteria. Notable exceptions include several clinically important Gram-positive organisms, including staphylococci and streptococci, which have retained the MVA pathway, and several obligate intracellular organisms, including rickettsiae and mycoplasmas, which have lost de novo isoprenoid metabolism altogether.

While most eukaryotes use the MVA pathway, phyla that have acquired a plastid organelle (either a true chloroplast, such as in plants, or a non-photosynthetic relic plastid) possess the eubacteria-like MEP pathway. In microbes, this includes the Apicomplexan protozoan pathogens *Toxoplasma gondii* (which causes toxoplasmosis) and the malaria-causing *Plasmodia* species, including the agent of severe malaria, *Plasmodium falciparum*. While plants contain both the MVA and MEP pathways, the Apicomplexans do not contain the MVA pathway and exclusively generate isoprenoids via the MEP pathway.

## Why Are Isoprenoids Essential in Pathogenic Microbes?

Isoprenoids are essential in all eubacteria in which they have been studied [7]–[9]. Genetic or chemical disruption of enzymes in this pathway is only possible with a redundant route for isoprenoid biosynthesis (either media supplementation with isoprenoids or transgenic expression of the MVA pathway). Isoprenoids are key to several core bacterial cellular functions. In eubacteria, there are at least three groups of isoprenoid compounds that appear to be essential—ubiquinones, menaquinones, and dolichols. Ubiquinones and menaquinones are electron carriers and major components of the aerobic and anaerobic respiratory chains, respectively [10]. Dolichols are long-chain, membrane-bound isoprenols, which are required for cell wall peptidoglycan synthesis [11]. Agents that block isoprenoid biosynthesis in bacteria therefore result in spheroplast formation and cell death [12].

The MEP pathway is also clearly essential in Apicoplexan eukaryotes, including the protozoan malaria parasite *P. falciparum* [13]–[15]. In contrast to eubacteria, it is not as clear why the malaria parasite needs isoprenoids. The clinical symptoms of malaria infection arise from the developmental cycle of the parasite within human erythrocytes. This privileged niche has resulted in several metabolic peculiarities. While cholesterol is essential to eukaryotic membrane stability, the parasite does not have de novo cholesterol biosynthesis and scavenges membrane cholesterol from host erythrocyte membranes [16]. The malaria parasite depends on glycolysis and does not use mitochondrial respiration for ATP production in the intraerythrocytic cycle [17]. In eukaryotes, dolichols are necessary for protein N-glycosylation, but *P. falciparum* produces severely truncated N-glycosyl groups that may not be required for protein function [18]. Finally, the malaria parasite makes plant-like signaling molecules (carotenoids), but these also do not have a known biological function [19]. The malaria parasite does have well-characterized protein prenyltransferases, which are expressed during the intraerythrocytic cycle [20], [21]. Protein prenylation is the isoprenyl modification of proteins, such as small GTPases. Either farnesyl (15 carbon) or geranylgeranyl (20 carbon) groups are attached to C-terminal cysteines by one of three prenyltransferases. Multiple classes of prenyltransferase inhibitors kill the malaria parasite, strongly suggesting that at least protein prenylation is an essential function of isoprenoid biosynthesis in malaria [20], [22].

## Why Is the MEP Pathway a Good Antimicrobial Drug Target?

The MEP pathway has a number of characteristics that make it a favorable target for antimicrobial drug development. Much like bacterial cell wall biosynthesis, which has been so successfully targeted by antibacterial agents, the MEP pathway is essential for microbial growth, and none of the enzymes of this pathway are present in mammalian cells. Validation of many of the properties of a new isoprenoid-inhibitor class of antibiotic has come from studies of the small phosphonic acid compound fosmidomycin. Fosmidomycin inhibits DXR (the first dedicated step in the MEP pathway) in multiple organisms [9], [15], [23]. It kills *E. coli* and *P. falciparum*, can treat malaria in field trials, and has been validated as an inhibitor of isoprenoid biosynthesis in vivo [14], [24]. Due to its highly charged structure, it has suboptimal pharmacokinetic properties, and is also excluded from and non-toxic to several organisms whose DXR enzyme is otherwise inhibited in vitro by fosmidomycin (including *T. gondii* and *Mycobacterium tuberculosis*) [9], [15]. Despite these challenges, fosmidomycin is currently in Phase II clinical trials in combination therapy to treat malaria, because of its favorable safety profile. Very high doses of fosmidomycin appear to be clinically safe—the oral mouse LD_50_ is a remarkable >11,000 mg/kg—presumably since there is no mammalian homolog to be inhibited [25].

Drug resistance has created an urgent need for new antimicrobial agents to combat Gram-negative bacteria, *M. tuberculosis*, and *P. falciparum*, which share the MEP pathway. New drugs that inhibit this pathway hold great promise as broad-spectrum agents, with potential appeal for commercially viable pharmaceutical development in the first-world as antibacterial agents (against resistant hospital-acquired infections, for example) and use in developing nations in combination therapies for malaria and tuberculosis.
